# Maladministration of Public Medical Affairs in the State of Texas*Read at meeting of the Texas State Medical Association, Waco, Texas, April 25, 1900.

**Published:** 1900-05

**Authors:** H. A. West

**Affiliations:** Galveston


### TEXAS MEDICAL JOURNAL.

AUSTIN, TEXAS.

ESTABLISHED JULY, 1885.

PUBLISHED MONTHLY.—SUBSCRIPTION $1.00 A YEAR.

VOL. XV. AUSTIN, MAY, 1900. No. 11.

### Original Contributions.

For Texas Medical Journal.

### Maladministration of Public Medical Affairs in the State of Texas.*

BY H. A. WEST, M. D., GALVESTON.

I have hesitated to write upon this subject for several reasons: First, because of my inability to do it justice. Second, because of the difficulty of discussing it from an impersonal standpoint. But having strong convictions as to the existence of radical defects in the management of public medical affairs in this State and of the fundamental and far-reaching evils resulting therefrom, I shall attempt, though in an imperfect way, to present my views for your careful consideration and such action as your wisdom may dictate.

First.—Let us examine the mode of administration of our hospitals for the insane.

Second.—The State Health Officer's functions, sins of omission and commission included.

Third.—The regulation of medical practice, and the extent of protection afforded the people by the present statutes upon the subject.

*Read at meeting of the Texas State Medical Association, Waco, Texas, April 25, 1900.

In regard to the administration of our hospitals for the insane, the people who pay the taxes for the maintenance of these institutions, the friends of the inmates and the unfortunates themselves have the right to demand that the enormous sums required for the erection of the necessary buildings and the support of the institutions shall be so expended as to obtain the best results. In other words, in assuming the care of its mentally defective population the State is obligated not to provide only a place of imprisonment for these people, to prevent injury to themselves or others, but to avail itself of all those means of cure and amelioration in the condition of the insane made accessible by modern enlightenment upon the subject. A prerequisite in the accomplishment of this end is to place at the head of our insane asylums medical men whose mental endowments, education and experience, along these special lines, enable them to successfully grapple with those recondite and difficult problems they are called upon to solve. If the State for any reason whatsoever, political favoritism, inadequate compensation, or short and uncertain tenure of office, fails to obtain and keep in its employ the best medical talent available within its borders, then the system of appointment and management it fatally and fundamentally defective, and should be corrected. I wish it distinctly understood that I am not attacking the present management of any one of our institutions. The only time I have visited one of our State asylums was a good many years ago in the city of Austin; the conditions then and there existing—arising from the ignorance and lack of experience upon the part of the superintendent—were abhorrent in the extreme, and afforded the best possible illustration of the evils of the system which could tolerate them. Let us investigate the *modus operandi* of appointment of the chief medical officers of our hospitals for the insane and the remuneration they receive. These officers are the appointees of the governor, who is elected every two years. It is safe to say that with every new governor there is a new batch of appointments. If by the grace of God, the manipulations of political wireworkers, to say nothing of the voice of the people, a governor is elected to the second term, then his appointees may hold over with him for two years longer. If our officer is a shrewd politician he may even stand a chance with the new governor, but if so misguided as to imagine that a strict attention to his business and an increasing ability to perform it will retain him in office, woe betide him; his official decapitation is sure to follow. After having sacrificed his private practice to serve the State, and just as he has commenced to acquire some valuable experience in this special line of work, he is ousted, and must perforce retire to the shades of private life and attempt to regain a practice long since scattered to the four winds of heaven. What compensation do these officers receive? Until the present year the superintendents were paid $2000 per annum, and were allowed board and lodging within the asylums—certainly not too much, taking into consideration the executive capacity, the technical skill, and the enormous responsibility involved. The legislature, however, at its last regular session, in the exercise of its profound wisdom and an economy which "saves at the spiggot and wastes at the bung hole," decreed that the officers should no longer be boarded at the expense of the State, without any proportionate increase of salary. The effect of this enactment cannot be otherwise than exceedingly detrimental, for together with the short and uncertain term of office, conditions prevail by which the applicants for these positions will be limited to that class of medical men who are either young and inexperienced, or to those who are not fitted either by natural endowments or education to render them successful in private practice or capable of filling such positions. It would be needless for me to present any argument to demonstrate the fact that the successful administration of our hospitals for the insane depends upon the possession of the requisite knowledge and skill upon the part of its executive head. The State, therefore, by the mode of appointment, by the short and uncertain tenure of office, and by its parsimony, is chargeable with contributory negligence; and by its failure to provide the means which will secure the best results in the treatment of the insane population stands indicted for maladministration.

Coming to a consideration now of the State Health Officer's functions and mode of administration, it is my intention to expose the evils of the system, and not to attack the present representative of the Health Department, for, however vulnerable the latter is, and however unfit he may have demonstrated himself to be, if he suits the governor who appointed him and the people he serves, I do not consider it my province to arraign him; and any criticism made as to his methods are rather intended to be illustrative of the evils I believe to exist. The Health Department of the State is composed of a State Health Officer and a few quarantine officers stationed at the principal ports and upon the Mexican border. So far as I know, their operations in the past have been confined to quarantine against three diseases, viz.: Small-pox, cholera and yellow fever. This season has been added, bubonic plague. One of the most important functions of the State is to avail itself of all those means for the conservation of the public health, which modern enlightenment, concerning the prevention of diseases, has placed at its disposal. That a State in this closing year of the nineteenth century should devote its entire energy to the quarantine of three or four infectious diseases and totally ignore many other of vastly more importance and other means of prevention and sanitation of far greater value is a condition so absurd and indefensible that a mere statement of the fact is sufficiently condemnatory. Preventive medicine is no longer a dream of the idealist, but a reality of transcending importance. The greatest accomplishments in the history of medical science have been made in the last three decades, and consist largely in the knowledge acquired of the causes of infectious diseases and methods of prevention. That a State should ignore such organization of its health department, as would enable it to put in operation modern means for the prevention of such scourges as tuberculosis, typhoid fever, diphtheria, scarlet fever, measles, malarial fever, dysentery and whooping cough, etc., is glaring evidence of maladministration. It is not contended that the entire work of prevention should be undertaken by the State, but it should take the initiative, following the lead of such States as Michigan, Massachusetts, New York, Pennsylvania, and others under the supervision of a State board of health, organization of county and municipal boards should be perfected in order to place in operation a comprehensive and modern system for the prevention of diseases.

A full statement of the foregoing and other facts bearing upon the subject was presented to the Legislature at its last regular session by an able and industrious committee from this body. Their failure to take action upon this and cognate matters was fully discussed at our last meeting. As heretofore mentioned, the entire efforts of the State and expenditure of money had been devoted to the maintenance of quarantine service against yellow fever, variola, cholera and bubonic plague. Before proceeding with an inquiry as to the *modus operandi* of this service it will be interesting to briefly discuss a few basic facts as to modern methods of quarantine, so as to contrast such methods with those in vogue under the present *regime* in Texas. It should be understood at the outset that quarantine is an evil, and that it may be an evil of enormous proportions in the paralysis of industry and commerce produced by it has been amply demonstrated time and again in the Southern States. An evil of such magnitude is only to be tolerated if it is capable of preventing a greater one, viz.: widespread pestilence and death. Granting that such is the case, the proposition cannot be disputed that the deleterious effects of quarantine are capable of enormous mitigation by an intelligent and modern mode of administration. It is not practicable within the limits allowed me to present a scintilla of the evidence going to show that quarantine, as conducted in this State, is not based upon intelligence or modern enlightenment, to say nothing of reason and common sense, I think, however, that I will be able to show that the methods in use are not only crude and inefficient, but that costly and unnecessary restrictions are placed upon commerce. Illustrative of this statement let us make a brief analysis of Rule No. 6, of Governor Sayers' quarantine proclamation, dated March 1st, 1899, which reads as follows: "Iron steamships arriving from ports north of 25 degrees north latitude not infected, with no cargo or passengers, or laden with such articles as cannot possibly be carriers of infection, with clean bills of health from last clearance and the clearance preceding the last, and in good sanitary condition at the time of arrival, may be permitted to enter, after being fumigated, without detention, if in the judgment of the local quarantine officer it is safe to do so." Iron steamships only, come within the provision of this ridiculous exemption. Wooden ships though, coming from non-infected ports, without cargo or passengers or laden with articles which cannot possibly be carriers of infection, and with clean bills of health, and in good sanitary condition, are subject to detention and the useless cost of fumigation. Again, iron steamships, from points north of 25 degrees north latitude not infected, *with* cargo or passengers or laden with such articles as cannot possibly be carriers of infection, with clean bills of health, etc., are subject to detention and fumigation. Still again, although a vessel may fulfill all the conditions of perfect safety, as specified in this rule, she is still subject to quarantine and disinfection at the discretion of the local quarantine officer. It is not necessary to mention instances of the application of Rule No. 6. That commerce is throttled by it, and that ports, when it is applied, are placed at enormous disadvantage in the competition for trade, goes without saying. To show further the workings of Texas quarantine, let me review the embargo against Galveston in 1898. It will be remembered that the State Health Officer was notified of a case of yellow fever at the U. S. barracks at Fort Point. The case was located about three miles from the city, and was as thoroughly isolated as if it had been upon the Dry Tortugas. Nevertheless, Galveston was at once placed under quarantine. The officials, however, of the G., C. & S. F. R. R. obtained permission from Dr. Blunt to remove a train load of their employes to Cleburne; this was done after the declaration of quarantine, and the ridiculous incident was heralded at Cleburne by the tootings of a brass band. Comment upon this remarkable proceeding would be a waste of words. Either the quarantine was unnecessary because Galveston was not an infected place, or, if the embargo was necessary, then the dispatch of a train load of people from it was an outrageous and dangerous act; and the perpetrators may accept either horn of the dilemma. The following statement is further illustrative of the *modus operandi* of Texas quarantine. It refers to a ship laden with coffee, part for New Orleans and part for Galveston. In response to urgent solicitations of the Galveston agent, Dr. Blunt replies, under date of March 16, 1900: "If the report and sanitary condition is good, I will allow her to unload by lighter, after ten days quarantine and thorough fumigation, and the ship will be then fumigated after the cargo is discharged. Then, if there is no sickness, she will be allowed to come to the wharf and take on cargo." It should be remembered that the enforcement of fumigation and re-fumigation, and ten day's detention, was made obligatory upon a ship whose health report and sanitary condition was good, and which had already undergone thorough disinfection at New Orleans. It is safe to say that no other port in the world labors under such restrictions, and, if allowed to continue, the fruitage of deep water at this port lies in the dim, distant future. If the State pretends to regulate its own quarantine, it should avail itself of all modern appliances for the purpose of doing so effectually, but with the least possible hindrance to commerce. The condition at Galveston, the chief port of the State, will show the lack of proper equipment. We have no hospitals for those sick with infections; no place of detention for those who have been exposed, and no adequate disinfecting plant.

In order to mitigate the evils of quarantine and to lessen the enormous losses and paralysis of industries incident to a general embargo, conventions have been held within the past three years in various Southern cities, notably at Atlanta and New Orleans. At these conventions were assembled the best representatives of the various interests involved, business men, railway officials, boards of health, physicians, sanitarians, and the U. S. Marine Hospital

Service. Resulting from their combined wisdom and application of scientific methods, plans have been devised whereby the evils of a general embargo might be averted. I cannot attempt to enumerate here the methods devised; they are embodied in the report of the Atlanta Convention, unanimously adopted April 12, 1898. The point I wish to make in this connection is that the State of Texas has not officially participated in any convention held for the purpose above mentioned, though urged to do so; but has stood aloof, and relied upon a policy of total non-intercourse. While such a policy appeals to a panic stricken and unreasoning mob, in my opinion it is a step backward; it fails to accomplish its purpose, and will, sooner or later, react disastrously upon those who attempt to enforce it. "There is more than one road leading to Rome." Merchants and passengers will find a way to circumvent the strictest shotgun quarantine; whereas, by camps of detention, careful inspection, classification of freight, disinfection, exemption as to trains bound for uninfected districts, etc., the rigors of quarantine may, to a great extent, be averted. Unless the States arrive speedily at some equitable adjustment of interstate quarantine, the Federal Government will be compelled to intervene, and by its power to regulate commerce between the States bring order out of the chaos heretofore existing.

Coming now to a brief consideration of the regulation of medical practice in this State, and the extent of protection afforded the people by the law now in force, the conditions resulting from the statutes now in operation cannot be better described than in the language of Dr. J. C. Loggins in his presidential address at Paris in 1897. He says, after giving a summary of the law, "that a critical examination of it is not necessary to show the defects that serve to make it far worse than useless, even absolutely hurtful both to the public and the medical profession of our State. That clause which admits registration of a diploma from some medical college, without enquiry as to the validity of the diploma, or standing of the college, has served to throw wide open the gateways of this State to every medical failure and fake in the Union. It is a well known fact that diplomas may be obtained from many so-called medical colleges as an article of commerce. That numbers of such diplomas have been purchased and registered in Texas, by men now engaged in practice, is equally well known, and serves as abundant proof of the fatal defect in our law." Dr. Loggins also called attention to another absurd and outrageous procedure of examining boards, viz.: the granting of license to practice to undergraduates.

I shall not discuss the rights of the educated and qualified practitioners to the protection of the law from competition by the traveling or resident quacks, mountebanks and imposters, with which this State is infested, but wish only to draw attention to what I believe has been the controlling idea with the legislatures of this State, which has heretofore influenced them to defeat every measure which has been offered to afford protection either to the people or to the medical profession. In my opinion, there is a deeply rooted idea in the mind of the average legislator that the various bills emanating from this body for the regulation of medical practice and the establishment of a State board of medical examiners were instigated mainly for the selfish motive of advancing the interests only of the medical profession. That the sole end we were endeavoring to accomplish was the organization of a great medical trust; whereas, the truth of the matter is that the public are far the greater sufferers from past and present conditions than ourselves. Let us glance for a moment at the facts. Let one of these perambulating, perennial quacks visit a town, his coming is heralded in the press with glittering promises to cure every ill to which human flesh is heir; its columns are filled with testimonials as to his miraculous cures; the hopes of the poor, misguided populace are raised to the highest possible pitch, they are ready and willing to make any sacrifice to secure the services of the so-called Divine Healer; women sell their jewelry; men pawn their household goods to obtain money and pour it into the lap of a miserable liar and cheat, who having stolen the livery of heaven to serve the devil in, masquerades in the guise of a physician. Who suffers most after such incursions, the physicians or the people? Again, let one of our district boards of examiners turn loose upon a community a graduate from one of the diploma mills, or a first or second course student to practice at the expense of the health and lives of an ignorant and confiding population. Who suffers most? When we succeed in educating our law-makers to a realization of the fact that the people, whose interests they are elected to subserve, are the chief sufferers from the present deplorable state of affairs, then and not before, will we succeed in our efforts to secure the enactment of an adequate law regulating the practice of medicine in the State of TEXAS.

GALVESTON, April 10, 1900.

